# Post-Mastectomy Direct-to-Implant Pre-Pectoral Breast Reconstruction With Polyurethane-Coated Implants: A Systematic Review of Efficacy and Safety

**DOI:** 10.1093/asjof/ojaf162

**Published:** 2025-12-04

**Authors:** Edoardo Caimi, Stefano Vaccari, Simone Furlan, Federico Corsi, Roberta Comunian, Riccardo Di Giuli, Valeria Bandi, Barbara Catania, Alessandra Veronesi, Francesco Klinger, Valeriano Vinci

## Abstract

Pre-pectoral (PP) direct-to-implant (DTI) breast reconstruction has gained popularity as a muscle-sparing alternative to subpectoral techniques. Polyurethane-coated implants, with their unique mechanical and biological properties, may further optimize outcomes by reducing capsular contracture and improving implant stability. This systematic review aimed to evaluate the safety, efficacy, and patient-reported outcomes of post-mastectomy PP DTI breast reconstruction using polyurethane-coated implants. A systematic search of PubMed, Embase, Scopus, and Cochrane Library databases was conducted in accordance with PRISMA guidelines. Data on study design, patient and surgical characteristics, complications, aesthetic outcomes, and patient satisfaction were extracted. Risk of bias was assessed using the ROBINS-I tool. Thirteen studies met inclusion criteria, comprising 1717 patients and 2502 breasts reconstructed with polyurethane-coated implants. Mean follow-up across included studies was 12.3 months (range 1-60 months). Mean implant size was 327 cc (range 160-680 cc). Across all studies, implant loss occurred in 1.0% of cases and infection in 0.9%. Capsular contracture with reported Baker grade III-IV cases occurred in 1.0% of cases within ≤12 months of follow-up, increasing to 12.3% in studies with ≥36 months’ follow-up. The most frequent aesthetic drawback was rippling or contour deformity, affecting 183 breasts (7.3%). Patient-reported satisfaction was consistently favorable: 5 studies using the BREAST-Q reported mean scores ranging from 72.6 to 78.3 for Satisfaction with Breasts and 79.9 to 84.6 for Psychosocial Well-Being. Pre-pectoral DTI reconstruction with polyurethane-coated implants is a safe and effective technique, associated with low complication rates and high patient satisfaction. Careful flap selection and adjunctive fat grafting optimize results. Prospective multicenter studies with long-term follow-up are warranted to confirm durability and refine patient selection criteria.

**Level of Evidence: 3 (Therapeutic):**

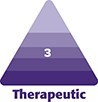

After non-melanoma skin cancer, breast cancer is the most common malignancy among women worldwide and remains a major global health burden. According to the most recent global estimates for 2024, breast cancer accounts for approximately 24% of all new cancer diagnoses in women and is responsible for 15% of cancer-related deaths in the female population.^1^ In parallel with oncologic advances, post-mastectomy breast reconstruction is now an integral element of comprehensive breast care.^2,3^ Contemporary US registry data from the American Society of Plastic Surgeons (ASPS) document 157,740 breast reconstructions performed in 2023, of which 117,512 were immediate procedures.^4^ Technique selection continues to favor prosthetic methods: tissue expander/implant (85,970) and direct-to-implant (DTI; 36,557) reconstructions as together comprised nearly 122,527 procedures in 2023, close to 78% of all reconstructions.^4-6^ On the other hand, autologous options for breast reconstruction have seen a slight increase in their use, with the deep inferior epigastric perforator flap being the most common modality for breast shape restoration.^7^

Within implant-based breast reconstruction, the pre-pectoral approach, where the implant is placed above the pectoralis major muscle, has gained substantial popularity in recent years.^4,8,9^ By preserving the pectoralis muscle, this technique avoids animation deformity, reduces postoperative pain, and maintains muscle function while allowing better control of breast shape.^10-12^ Comparative studies and meta-analyses have consistently shown that, in appropriately selected patients, complication rates are comparable to those of the subpectoral approach, with some reports suggesting advantages in terms of reduced mastectomy flap necrosis and improved patient satisfaction.^8,9,13^ Long-term follow-up studies have also confirmed oncologic safety, with recurrence rates and survival outcomes similar to traditional methods.^14^ In the pre-pectoral setting, implant–tissue interface mechanics are particularly important, and polyurethane (PU) foam-coated implants have been shown to promote tissue adherence and alter capsule formation, lowering the risk of implant malposition and capsular contracture.^15-17^ Their comparatively low rate of capsular contracture relates to their distinctive surface that sets them apart from traditional silicone or saline devices.^18^ The unique degradation process of the PU particles coating the implant shell is the mechanism behind their biological properties. As these particles gradually break down, they stimulate giant cells and macrophages to phagocytose the fragments, initiating a granulomatous foreign body–type reaction.^19^ This reaction leads to the development of numerous discrete “microcapsules” around the implant, which remain separate rather than merging into a single fibrous capsule. This microencapsulation pattern is thought to limit capsular tightening and has supported the increasing adoption of PU-coated implants in reconstructive breast surgery.^16,17^

Despite these favorable trends, the evidence in the literature regarding the use of pre-pectoral DTI with PU-coated implants post-mastectomy breast reconstruction remains dispersed across small, often single-center studies with heterogeneous reporting and follow-up. Given the widespread clinical adoption of the pre-pectoral approach and the device-specific mechanics of PU coatings, a rigorous synthesis focused on efficacy and safety is timely. The present systematic review aims to consolidate contemporary data to inform patient selection, surgical planning, and device choice in immediate pre-pectoral DTI reconstruction with PU-coated breast implants.

## METHODS

### Protocol Registration

A systematic review of the literature regarding the efficacy and safety of pre-pectoral PU-coated breast implants adopted as DTI post-mastectomy breast reconstruction technique was conducted following the guidelines outlined in the Preferred Reporting Items for Systematic Reviews and Meta-Analyses (PRISMA) protocol (Supplemental Table 1).^20^ The protocol was registered prospectively on the PROSPERO database (registration ID: CRD420251123016; available from: https://www.crd.york.ac.uk/PROSPERO/view/CRD420251123016).

### Study Selection

Inclusion criteria for this systematic review included all studies involving adult patients (≥18 years) undergoing immediate DTI pre-pectoral breast reconstruction following therapeutic or prophylactic nipple-sparing mastectomy (NSM), skin-sparing mastectomy (SSM), or skin-reducing mastectomy (SRM) in which PU-covered silicone implants were used. Eligible studies were required to report at least one relevant postoperative outcome, including capsular contracture, infection, seroma, implant loss, wound healing problems, rippling, patient-reported satisfaction, aesthetic results, or reoperation rates.

Eligible study designs included comparative cohorts, randomized controlled trials, prospective or retrospective cohort studies, and single-arm observational studies (retrospective or prospective) provided they enrolled ≥10 reconstructed breasts and reported on our outcomes of interest. Only English studies published in peer-reviewed journals with full-text availability were considered. No restrictions were placed on the year of publication to limit selection bias.

Exclusion criteria included non-human or cadaveric studies, review articles, conference abstracts without full data, single case reports, and studies not reporting relevant postoperative outcomes. Studies were also excluded if they did not clearly describe the surgical procedure, did not involve immediate pre-pectoral reconstruction, or did not use PU-covered implants. In cases of duplicate publications reporting on the same patient cohort, the most complete or recent dataset was included.

### Search Strategy and Screening

On August 10, 2025, a comprehensive literature search was conducted for articles published up to the day of search in the following databases: PubMed, Embase, Cochrane, and Scopus. The search aimed to identify studies evaluating and analyzing the outcomes of DTI pre-pectoral PU-coated breast implants in female patients who underwent mastectomy, as their surgical reconstructive technique. The following search string combining Medical Subject Headings (MeSH) terms and relevant keywords was used: ((“mastectomy” OR “nipple-sparing mastectomy” OR “conservative mastectomy”) AND (“immediate reconstruction” OR “immediate breast reconstruction” OR “direct-to-implant” OR “first stage breast reconstruction”) AND (“pre-pectoral implants” OR “pre-pectoral breast reconstruction” OR “pre-pectoral reconstruction”) AND (“polyurethane-coated implants” OR “polyurethane breast implants” OR “polyurethane-coated silicone implants”)). Vocabulary and search syntax were adapted as appropriate for each database to ensure a sensitive and specific search strategy.

The initial assessment of search results was performed by one author (E.C., plastic surgery resident), who identified and removed duplicate records. Following duplicate removal, 3 authors (E.C., S.V., S.F., all plastic surgery residents) independently screened the remaining articles by title and abstract to assess potential eligibility. Full-text screening of selected articles was subsequently conducted by the same 3 reviewers to confirm inclusion. Any disagreements or uncertainties during the selection process were resolved through discussion with a fourth reviewer (V.V., senior plastic surgeon).

### Data Extraction and Statistical Analysis

Data extraction was performed independently by 2 authors (E.C. and F.C., all plastic surgery residents) using a standardized, pre-piloted Microsoft Excel (Microsoft Corporation, Redmond, WA) sheet. For each included study, information was collected on study characteristics (first author, year of publication, country, study design), patient demographics (number of patients and breasts, mean or median age, body mass index, smoking status, comorbidities), surgical details (mastectomy type, use of acellular dermal matrix [AMD] or mesh, implant size and shape, laterality, and receipt of neo-adjuvant or adjuvant radiotherapy or chemotherapy), and follow-up duration. Primary outcomes included the incidence of capsular contracture, infection, seroma, implant loss, wound healing problems, and rippling. Secondary outcomes comprised patient-reported satisfaction, aesthetic results, and reoperation rates. Where outcome definitions or time points varied between studies, these were recorded exactly as reported in the original publication. Any missing or unclear data were noted, and Supplementary material or direct author contact was used, when possible, to clarify them. Discrepancies in data extraction were resolved through discussion and consensus, with arbitration by a third reviewer (V.V., senior plastic surgeon) when necessary. Descriptive statistics were used. Where comparable data were available, complication rates and selected outcomes were pooled across studies and expressed as total counts and proportions relative to the pooled sample size. Continuous variables were summarized as means and ranges when reported in the primary studies. No inferential or comparative statistical testing was performed.

### Bias Evaluation

The risk of bias of included non-randomized studies was evaluated by the Risk of Bias in Nonrandomized Studies of Interventions (ROBINS-I) tool.^21^ This instrument evaluates 7 domains of potential bias and assigns each a judgment of low, moderate, serious, or critical risk. An overall risk of bias rating is then determined based on the highest level of bias observed across the domains. Two reviewers (E.C. and R.C., all plastic surgery residents) independently evaluated each study. Discrepancies in scoring were resolved through discussion, and when consensus could not be reached, a third reviewer (F.K., senior plastic surgeon) served as the arbitrator. The results of the quality assessment are presented in tabular form in the “Results” section.

## RESULTS

### Literature Search

**Figure 1.**
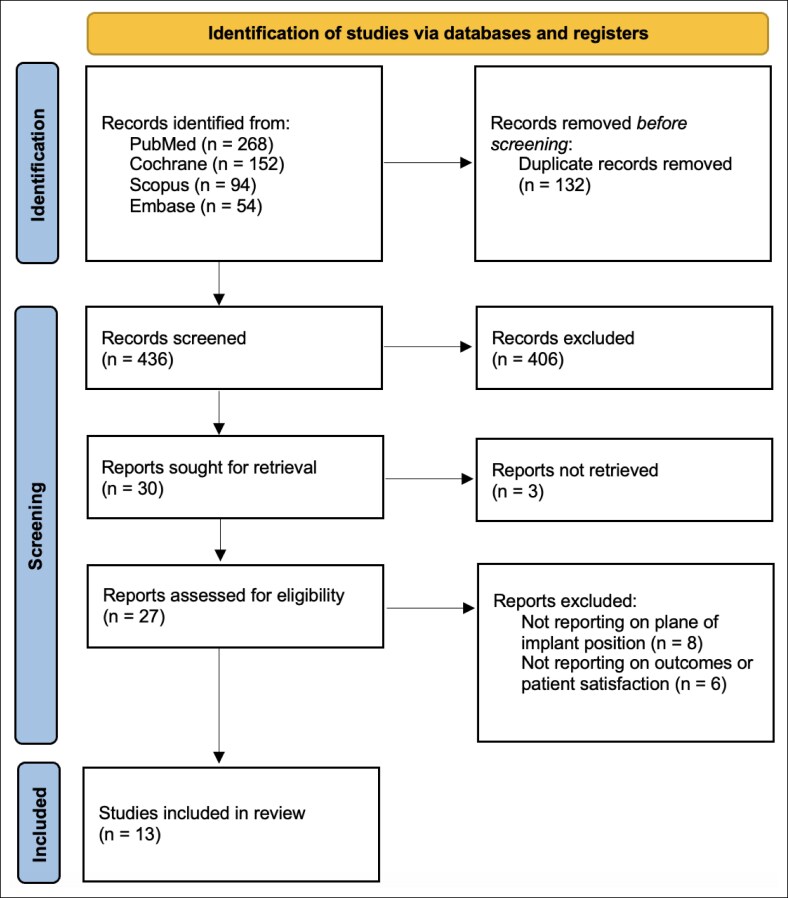
Flow chart of study search and selection according to Preferred Reporting Items for Systematic Reviews and Meta-Analyses (PRISMA).

The search strategy yielded a total of 568 articles, including 268 from PubMed, 152 from Cochrane and 94 from Scopus, and 54 from Embase. One hundred and thirty-two of these articles were duplicates and were promptly removed. The remaining 436 articles were evaluated for their inclusion in the study. A total of 406 articles were excluded during the initial screening process for not meeting the inclusion criteria. We sought to retrieve 30 reports, with 3 reports not retrieved. The remaining 27 reports were assessed for eligibility. After full-text review, 14 reports were excluded. Ultimately, 13 studies^19,22-33^ met the inclusion criteria and were included in the final systematic review. The detailed flow chart for study selection according to PRISMA guidelines is presented in Figure 1.

### Study Characteristics and Quality Assessment

Thirteen studies published between 2019 and 2025 were included, comprising a total of 1717 patients and 2502 breasts undergoing pre-pectoral DTI breast reconstruction with PU-coated implants. Most studies were conducted in Europe, particularly Italy (*n* = 10; 76.92%),^19,22,23,25-28,30,32,33^ with additional cohorts from Belgium (*n* = 1; 7.69%),^24^ Spain (*n* = 1; 7.69%),^29^ Portugal (*n* = 1; 7.69%),^31^ and the United Kingdom (*n* = 1; 7.69%).^26^ The majority were retrospective cohort studies,^19,22,25-28,30,32,33^ while 2 prospective observational studies^24,29^ and 1 comparative cross-sectional analysis^31^ were also identified. Study periods ranged from 2015 to 2024, reflecting the growing adoption of this technique in recent years. Sample sizes varied widely, from a minimum of 21 patients^23^ to a maximum of 453.^27^ The mean age of participants across studies was 47.76 years and ranged from 21^30^ to 78^26^ years. Importantly, the mean follow-up duration was 12.3 months with a range of a minimum of 1 month^25^ follow-up to a maximum of 60.^26^ Table 1 depicts all the characteristics of the included studies.

**Table 1.** Characteristics of Included Studies

Author (date of publication)	Country	Study design	Period of study	Pre-pectoral PUCI patients	Pre-pectoral PUCI breasts	Mean age in years (SD) [range]^a^	Mean follow-up in months (SD) [range]^a^
de Vita et al (2019)^23^	Italy	Retrospective cohort	December 2017-April 2018	21	34	42 [31-48]	4 [2-6]
Coyette et al (2021)^24^	Belgium	Prospective cohort study	January 2018-December 2019	57	74	50 [29-75]	18.5 [12-24]
Scardina et al (2021)^25^	Italy	Retrospective cohort	January 2018-April 2021	209	269	47 [25-73]	14 [1-40]
Salgarello et al (2021)^26^	Italy, UK	Retrospective cohort	January 2015-December 2019	266	428	49 [27-78]	26 [6-60]
de Vita et al (2022)^27^	Italy	Retrospective Cohort	December 2017-June 2021	453	784	43.5 [29-65]	NR [6-42]
Pagliara et al (2023)^28^	Italy	Retrospective cohort	January 2020-April 2021	100	124	46.49 (10.51)	12
Nebril et al (2023)^29^	Spain	Observational prospective	November 2018-October 2021	102	159	46.8 (7.8)	11
Catanuto et al (2024)^30^	Italy	Retrospective cohort	February 2019-March 2021	63	74	49 [21-69]	NR
Correia-Pinto et al (2024)^31^	Portugal	Comparative cross-sectional	June 2018-June 2022	59	44	54.1 (8.7)	16 [6-24]
Lembo et al (2024)^32^	Italy	Retrospective cohort	January 2018-June 2021	21	36	47 [30-66]	19.3 (2.8) [12-53]
Lisa et al (2025)^19^	Italy	Retrospective cohort	March 2020-December 2022	250	317	50.5 (10.9)	NR
Salgarello et al (July 2025)^22^	Italy	Retrospective cohort	December 2018-May 2024	62	89	49	NR
Salgarello et al (August 2025)^33^	Italy	Retrospective cohort	April 2015-October 2019	54	70	47.1 [42.5-51.5]	40 [12-60]
Overall				1717	2502	47.76 [21-78]	12.3 [1-60]

NR, not reported; PUCI, polyurethane-coated breast implants; SD, standard deviation. ^a^Data were extracted according to the values reported in the included articles. When studies provided only the standard deviation or the range, that value alone was reported. When both SD and range were available, both were included in the tables.

The methodological quality of the included studies was assessed using the ROBINS-I tool.^21^ Across all 13 included studies, the overall risk of bias was judged as moderate^23-28,30,31^ or low-to-moderate.^19,22,29,32,33^ The most frequent concerns were related to confounding, since most studies were retrospective single-arm cohorts without multivariable adjustment, and to reporting bias, as many studies did not include all fundamental parameters regarding the patient population and outcomes. Nevertheless, interventions were consistently well-defined, perioperative protocols were standardized, and outcome measures were reliable. No study was judged to be at serious or critical risk of bias. Table 2 summarizes the individual study characteristics regarding bias risk assessment using the ROBINS-I tool.

**Table 2.** Quality Assessment According to the Risk of Bias in Nonrandomized Studies-I (ROBINS-I) Tool

Author (date of publication)	Confounding	Selection	Classification	Deviations	Missing data	Outcome measurement	Reporting	Overall risk^a^
de Vita et al (2019)^23^	Moderate	Moderate	Low	Moderate	Moderate	Moderate	Moderate	Moderate
Coyette et al (2021)^24^	Moderate	Moderate	Low	Moderate	Moderate	Moderate	Moderate	Moderate
Scardina et al (2021)^25^	Moderate	Moderate	Low	Low	Moderate	Moderate	Moderate	Moderate
Salgarello et al (2021)^26^	Moderate	Moderate	Low	Low	Moderate	Moderate	Moderate	Moderate
de Vita et al (2022)^27^	Moderate	Moderate	Low	Low	Moderate	Moderate	Moderate	Moderate
Pagliara et al (2023)^28^	Moderate	Moderate	Low	Low	Moderate	Moderate	Moderate	Moderate
Nebril et al (2023)^29^	Moderate	Low	Low	Low	Moderate	Low	Moderate	Low-moderate
Catanuto et al (2024)^30^	Moderate	Moderate	Low	Low	Moderate	Moderate	Moderate	Moderate
Correia-Pinto et al (2024)^31^	Moderate	Moderate	Low	Low	Moderate	Moderate	Moderate	Moderate
Lembo et al (2024)^32^	Moderate	Low	Low	Low	Moderate	Moderate	Moderate	Low-moderate
Lisa et al (2025)^19^	Moderate	Moderate	Low	Low	Moderate	Low	Low	Low-moderate
Salgarello et al (July 2025)^22^	Moderate	Moderate	Low	Low	Low	Low	Moderate	Low-moderate
Salgarello et al (August 2025)^33^	Moderate	Moderate	Low	Low	Low	Low	Moderate	Low-moderate

^a^Low risk of bias: study is comparable to a well performed randomized trial within that domain. Moderate risk of bias: study is sound for a non-randomized study but is not considered comparable to a well performed randomized trial within that domain. Serious risk of bias: study has some important problems within that domain. Critical risk of bias: the study is too problematic in this domain to provide any useful evidence on the effects of intervention.

### Baseline Sample Characteristics

Across the 13 included studies, with a total of 1717 patients, reporting of baseline variables was heterogeneous, with several cohorts excluding higher-risk candidates such as diabetics^23,28^ or those exposed to neo-adjuvant or adjuvant treatment,^28^ and many series not reporting all items. Where available, smokers accounted for 74 patients (4.3%), diabetes for 21 (1.2%), and hypertension for 51 (3.0%) of the pooled cohort. These figures likely underestimate true prevalence given systematic exclusions and missing data. Regarding oncologic profile, among studies that specified tumor type, invasive ductal carcinoma (IDC) predominated (*n* = 476; 27.7%), with additional cases of DCIS (*n* = 52; 3.0%) and invasive lobular carcinoma (*n* = 83; 4.8%) reported; several series also included risk-reducing/prophylactic indications captured within “other” categories (*n* = 71; 4.1%). Stage distribution where stated was skewed toward early disease (T1: 242 cases, 14.1%; T2: 126 cases, 7.3%), with very few T3 tumors (*n* = 17; 1.0%) and a subset of Tis lesions (*n* = 102; 5.9%). Systemic therapies were variably used and inconsistently reported across studies; pooled counts indicate neoadjuvant chemotherapy in 217 patients (12.6%), adjuvant chemotherapy in 74 (4.3%), and adjuvant radiotherapy in 141 (8.2%). Detailed baseline characteristics are presented in Table 3.

**Table 3.** Baseline Characteristics of Patients in the Included Studies

Author (date of publication)	Pre-pectoral PUCI patients	Total smokers (%)	Total comorbidities present (%)	Diabetes (%)	Hypertension (%)	Breast cancer type (%)				Breast cancer TNM (%)					Neo-adjuvant chemotherapy (%)	Adjuvant chemotherapy (%)	Adjuvant radiotherapy (%)
						IDC	DCIS	ILC	Other	Tis	Tx	T1	T2	T3			
de Vita et al (2019)^23^	21	0 (0)	NR	0; diabetes was considered an exclusion criteria	NR	NR	NR	NR	NR	NR	NR	T1 only (amount NR)	NR	NR	NR	NR	NR
Coyette et al (2021)^24^	57	6 (10.53)	NR	NR	NR	NR	NR	NR	NR	NR	NR	NR	NR	NR	8 (14.04)	NR	15 (26.32)
Scardina et al (2021)^25^	209	NR	NR	NR	NR	152 (72.73)	27 (12.92)	22 (10.53)	8 (3.83)	27 (12.91)	39 (18.6)	98 (46.89)	38 (18.18)	7 (3.35)	81 (38.76)	NR	NA
Salgarello et al (2021)^26^	266	NR	NR	NR	NR	NR	NR	NR	NR	NR	NR	NR	NR	NR	NR	NR	NR
de Vita et al (2022)^27^	453	0 (0)	NR	0 (0)	NR	Lobular carcinoma, early-stage invasive carcinoma				NR	NR	T1 only (amount NR)	NR	NR	NR	NR	NR
Pagliara et al (2023)^28^	100	NR	NR	0; diabetes was considered an exclusion criteria	NR	54 (54.0)	14 (14.0)	8 (8.0)	NR	NR	NR	NR	NR	NR	0; part of the exclusion criteria	NR	0; part of the exclusion criteria
Nebril et al (2023)^29^	102	NR	NR	NR	NR	60 (58.82)	11 (10.78)	11 (10.78)	NR	10 (9.80)	10 (9.80)	26 (25.49)	22 (21.57)	4 (3.92)	31 (30.5)	NR	40 (39.22)
Catanuto et al (2024)^30^	63	6 (9.52)	8 (12.70)	8 (12.69)	0 (0)	NR	NR	NR	NR	NR	NR	NR	NR	NR	15 (23.81)	21 (33.33)	11 (17.46)
Correia-Pinto et al (2024)^31^	59	15 (25.42)	NR	4 (6.78)	23 (38.98)	NR	NR	NR	NR	NR	NR	NR	NR	NR	NR	NR	3 (5.08)
Lembo et al (2024)^32^	21	5 (23.81)	15 (71.43)	3 (14.29)	5 (23.81)	NR	NR	NR	NR	NR	NR	NR	NR	NR	1 (4.76)	1 (4.76)	NR
Lisa et al (2025)^19^	250	27 (10.8)	46 (18.4)	3 (1.20)	17 (6.80)	210 (84.0)	NR	42 (16.8)	65 (26.0)	65 (20.2)	24 (7.60)	118 (37.2)	66 (20.8)	6 (1.90)	40 (16.0)	48 (19.2)	50 (20.0)
Salgarello et al (July 2025)^22^	62	11 (17.74)	14 (22.58)	1 (1.61)	6 (9.68)	NR	NR	NR	NR	NR	NR	NR	NR	NR	11 (17.74)	NR	NR
Salgarello et al (August 2025)^33^	54	4 (7.41)	2 (3.70)	2 (3.70)	NR	NR	NR	NR	NR	NR	NR	NR	NR	NR	30 (55.5)	4 (7.41)	22 (40.74)
Overall	1717	74 (4.31)	85 (4.95)	21 (1.22)	51 (2.97)	476 (27.72)	52 (3.03)	83 (4.83)	71 (4.14)	102 (5.94)	73 (4.25)	242 (14.09)	126 (7.33)	17 (0.99)	217 (12.64)	74 (4.31)	141 (8.21)

DCIS, ductal carcinoma in situ; IDC, invasive ductal carcinoma; ILC, invasive lobular carcinoma; NR, not reported; PUCI, polyurethane-coated breast implants.

### Surgical Characteristics and Perioperative Management of the Patient Sample

The number of surgeons varied considerably, from single-surgeon cases^23^ to multi-surgeon or institutional teams.^29-31^

All included studies described DTI pre-pectoral reconstruction using PU-coated silicone implants, specifically Polytech (Microthane Sublime Line, Dieburg, Germany). The majority of procedures followed NSM^19,22-31,33^ (1950 total reported procedures; 94.29%), although several cohorts also included SSM^19,22,29-31,33^ (108 total reported procedures; 5.22%) or SRM^22,32^ (10 total reported procedures; 0.49%). The mean implant volume across studies was 326.9 cc (range 160-680 cc), with larger implants more frequently used by Salgarello et al to treat large and ptotic breasts.^33^

Drain protocols varied: Salgarello et al^22^ and Scardina et al^25^ used 2 Jackson–Pratt drains per breast placed in the pre-pectoral plane; removal criteria were output-based in both (first day with <25-40 mL/day). In contrast, Lembo et al^32^ and Lisa et al^19^ routinely used Blake-type (Ethicon, Johnson-Johnson, Raritan, NJ) drains. Other author reported the use of surgical drains positioned in the operated plane but did not specify which was used.

Several studies integrated objective intraoperative flap assessment. Four studies^22,26,28,33^ provided detailed protocols for intraoperative evaluation of mastectomy flap viability using both thickness measurement and indocyanine green (ICG) angiography. Pagliara et al^28^ measured flap thickness intraoperatively at 9 points by ultrasound, reporting a mean thickness of 1.3 ± 0.5 cm in patients without ischemic complications vs 0.8 ± 0.3 cm in those with complications; a flap thickness ratio < 0.5 was strongly predictive of necrosis. Adequate perfusion on ICG angiography was defined as complete flap enhancement within 120 seconds after administration of 0.25 mg/kg ICG. Salgarello et al^26^ also adopted a dual-assessment protocol, considering ≥0.8 cm as the minimal safe flap thickness, with thicker flaps (>1 cm) correlating with improved aesthetic outcomes and fewer cases of rippling. Similarly, ICG angiography confirmed perfusion adequacy when uniform enhancement occurred within 120 seconds of injection. Finally, in a cohort of large and ptotic breasts, Salgarello et al^33^ applied even stricter criteria, excluding patients with flap thickness <1 cm or with non-uniform ICG enhancement. The mean flap thickness in this series was 1.1 cm, and patients selected under these criteria achieved low rates of ischemic complications despite challenging anatomy. Lisa et al^19^ used a reproducible threshold of ≥0.8-cm preoperative “pinch test” at the upper breast pole. Earlier reports relied primarily on intraoperative and clinical judgment.^23,24,27,29,32^

Table 4 depicts all of the surgical details extracted from the included studies.

**Table 4.** Surgical Characteristics of Patients in the Included Studies

Author (date of publication)	Number of surgeons	Mastectomy type	Indication for mastectomy (number of breasts)	Type of implant positioned	Mean implant volume (SD) [range]	Mastectomy flap evaluation	Postoperative drains	Number of days of drainage [range]
de Vita et al (2019)^23^	1	NSM	Prophylactic (26) and therapeutic (8)	Polytech Microthane^a^	395 cc [350-495]	Clinical	Yes, 1 drain	8 [6-10]
Coyette et al (2021)^24^	NR	NSM	Prophylactic (15) and therapeutic (42)	Polytech Microthane^a^	335 cc [160-500]	Clinical	Yes, 1 drain	NR
Scardina et al (2021)^25^	NR	NSM	NR	Polytech Microthane^a^	NR	NR	Yes, 2 Jackson–Pratt drains	NR
Salgarello et al (2021)^26^	NR	NSM	Prophylactic (NR) and therapeutic (NR)	Polytech Microthane^a^	345 cc [165-520]	ICG angiography (adequate perfusion if uniform enhancement within 120 seconds) and intraoperative US measurement (≥0.8 cm as the minimal safe flap thickness)	Yes, no other detail	NR
de Vita et al (2022)^27^	NR	NSM	Prophylactic (NR) and therapeutic (NR)	Polytech SublimeLine Microthane^a^	420 cc [350-535]	Clinical	Yes, no other detail	9 [6-10]
Pagliara et al (2023)^28^	NR	NSM	Prophylactic (NR) and therapeutic (NR)	Polytech SublimeLine Microthane^a^	NR	ICG angiography (adequate perfusion if uniform enhancement within 120 seconds) and intraoperative US measurement at 9 points (mean thickness of 0.8 ± 0.3 cm was associated with complications)	Yes, no other detail	NR
Nebril et al (2023)^29^	5	SSM, NSM	Prophylactic (34) and therapeutic (125)	Polytech Microthane^a^	290,6 cc (230,3)	Clinical: Rancati classification (“Breast tissue coverage classification”))	Yes, no other detail	NR
Catanuto et al (2024)^30^	4	NSM, SSM	NR	Polytech Microthane^a^	NR	NR	Yes, no other detail	NR
Correia-Pinto et al (2024)^31^	2	SSM, NSM, SRM	Prophylactic (12) and therapeutic (32)	Polytech Microthane, Replicon or Opticon^a^	315 cc [275-395]	NR	NR	NR
Lembo et al (2024)^32^	NR	SRM	Prophylactic (30) and therapeutic (6)	Polytech Microthane^a^	445 cc [380-550]	Clinical: color, capillary refill, dermal bleeding	Yes, a 10 F drainage (Blake^b^)	NR
Lisa et al (2025)^19^	NR	NSM, SSM	Prophylactic (39) and therapeutic (278)	Polytech Microthane^a^	429 cc (114)	Clinical: preoperative “pinch test” (threshold of ≥0.8 cm at the upper breast pole)	Yes, drainage (Blake-type^b^)	10.7 [6-12]
Salgarello et al (July 2025)^22^	NR	NSM, SSM	NR	Polytech Microthane Opticon^a^	490 cc [335-680]	ICG angiography and intraoperative measurement (exclusion of all patients with non-uniform ICG angiography patter and flap thickness <1 cm)	Yes, no other detail	NR
Salgarello et al (August 2025)^33^	NR	NSM, SRM, SSM	NR	Polytech Microthane^a^	295 cc [235-400]	Clinical: intraoperative assessment at 4 distinct points (exclusion of all patients with flap thickness <1 cm) and ICG angiography (decision based on uniformity)	Yes, 2 Jackson–Pratt drains	NR

ICG, indocyanine green; NSM, nipple-spearing mastectomy; NR, not reported; SRM, skin-reducing mastectomy; SSM, skin-spearing mastectomy; US, ultrasound. aLine, Dieburg, Germany. bEthicon, Johnson-Johnson, Unites States.

### Postoperative Complications

Complications were variably reported across studies, with a pooled incidence of 391 events (15.6%) among 2502 breasts reconstructed with PU-coated implants. The most frequent complication was rippling or implant visibility, described in 183 breasts (7.3%). Rates were particularly high in larger cohorts such as Lisa et al^19^ (14.5%) and Salgarello et al^26^ (21.4%), but were rarely reported in early series like de Vita et al^23^ or Scardina et al.^25^ Skin or NAC-related complications were also observed, with partial flap or nipple–areola necrosis in 55 cases (2.2%) and full-thickness flap necrosis in 17 cases (0.7%). Implant-related events included implant loss in 24 cases (1.0%), most frequently in Catanuto et al^30^ (6.8%) and Lisa et al^19^ (3.5%). Capsular contracture was infrequent within the reported follow-up (26 cases; 1.0%). When stratified by follow-up duration, rates were 1.0% (136 total breasts) in studies with ≤12 months of follow-up, 4.5% (547 total breasts) in those with 12-24 months, and 12.3% (498 total breasts) in studies with ≥36 months. Notably, Salgarello et al^22^ reported a higher rate (35.7%) at 3-5 years, reflecting its long-term comparative cohort, which had a mean follow-up of 40 months and extended up to 60 months.

Other complications were less frequent: seroma (0.9%), infection (0.9%), wound dehiscence (0.6%), and hematoma (0.3%). The highest infection rates were seen in Nebril et al^29^ (2.5%) and Catanuto et al^30^ (5.4%). A detailed summary of postoperative complication is reported in Table 5.

**Table 5.** Complications Associated With Post-mastectomy Immediate Breast Reconstruction With Pre-pectoral Polyurethane-Coated Implants Reported by the Included Studies

Author (date of publication)	Pre-pectoral PUCI patients	Pre-pectoral PUCI breasts	Total complications (%)	Rippling (%)	Partial flap or NAC necrosis (%)	Grade III/IV capsular contracture (%)	Implant loss (%)	Seroma (%)	Implant infection (%)	Full-thickness mastectomy flap necrosis (%)	Wound dehiscence (%)	Hematoma (%)	Bleeding (%)
de Vita et al (2019)^23^	21	34	1 (2.94)	0 (0)	0 (0)	NR	0 (0)	0 (0)	0 (0)	0 (0)	0 (0)	0 (0)	0 (0)
Coyette et al (2021)^24^	57	74	10 (13.51)	2 (3.51)	1 (1.35)	1 (1.35)	1 (1.35)	2 (3.51)	1 (1.35)	0 (0)	1 (1.35)	1 (1.35)	0 (0)
Scardina et al (2021)^25^	209	269	5 (1.86)	0 (0)	0 (0)	0 (0)	3 (1.12)	0 (0)	0 (0)	2 (0.74)	0 (0)	0 (0)	0 (0)
Salgarello et al (2021)^26^	266	428	51 (11.91)	12 (2.80)	39 (9.11)	0 (0)	0 (0)	0 (0)	0 (0)	0 (0)	0 (0)	0 (0)	0 (0)
de Vita et al (2022)^27^	453	784	82 (10.46)	82 (10.46)	NR	0 (0)	0 (0)	0 (0)	0 (0)	0 (0)	0 (0)	0 (0)	0 (0)
Pagliara et al (2023)^28^	100	124	21 (16.94)	14 (11.29)	4 (3.23)	NR	NR	NR	NR	3 (2.42)	NR	NR	NR
Nebril et al (2023)^29^	102	159	18 (11.32)	NR	3 (1.89)	NR	4 (2.52)	4 (2.52)	NR	0 (0)	4 (2.52)	2 (1.26)	1 (0.98)
Catanuto et al (2024)^30^	63	74	19 (25.68)	NR	0 (0)	0 (0)	5 (6.76)	4 (5.41)	3 (4.05)	5 (6.76)	0 (0)	2 (2.70)	0 (0)
Correia-Pinto et al (2024)^31^	59	44	3 (6.82)	NR	2 (4.55)	0 (0)	NR	0 (0)	0 (0)	NR	NR	1 (2.27)	NR
Lembo et al (2024)^32^	21	36	2 (5.55)	0 (0)	1 (2.78)	0 (0)	0 (0)	0 (0)	0 (0)	0 (0)	1 (2.78)	0 (0)	0 (0)
Lisa et al (2025)^19^	250	317	108 (34.07)	46 (14.51)	0 (0)	24 (7.57)	11 (3.47)	8 (2.52)	8 (2.52)	5 (1.58)	2 (0.63)	2 (0.63)	5 (1.58)
Salgarello et al (July 2025)^22^	62	89	20 (22.47)	12 (13.48)	NR	1 (1.12)	NR	2 (2.25)	1 (1.12)	NR	4 (4.49)	NR	NR
Salgarello et al (August 2025)^33^	54	70	51 (72.86)	15 (21.43)	5 (7.14)	25 (35.71)	0 (0)	2 (2.86)	4 (5.71)	0 (0)	0 (0)	0 (0)	0 (0)
Overall	1717	2502	391 (15.63)	183 (7.31)	55 (2.19)	26 (1.04)	24 (0.96)	22 (0.88)	17 (0.68)	15 (0.59)	12 (0.48)	7 (0.28)	6 (0.24)

NR, not reported; PUCI, polyurethane-coated breast implants.

### Aesthetic Outcomes and Patient Satisfaction

Eight of the included studies^25,26,28,30-33^ reported patient-reported or aesthetic outcomes, though methodologies varied. Five cohorts^26,28,31-33^ used the BREAST-Q validated tool before and after surgery. Across these studies, mean Satisfaction with Breasts scores ranged from 72.6 to 78.3, Psychosocial Well-Being from 79.9 to 84.6, and Sexual Well-Being from 67.4 to 72.8, reflecting consistently high satisfaction levels. Predictors of superior scores included thicker mastectomy flaps^26,28^ while Lembo et al^32^ identified diabetes as an independent predictor of lower scores.

Beyond patient-reported outcome measures (PROMs), Scardina et al^25^ employed a custom survey, confirming favorable outcomes though lacking external validation. Objective assessments were performed in Salgarello et al,^22^ where blinded surgeon panels using structured aesthetic grading demonstrated that PU reconstructions achieved significantly higher overall aesthetic scores (odds ratio: 0.372; *P* = .02), better symmetry and breast position (odds ratio: 0.676; *P* = .03), and fewer contour deformities compared to ADM-based reconstructions, with lower rates of capsular contracture at long-term follow-up. Catanuto et al,^30^ which used BCCT.core software for objective cosmetic evaluation that reported excellent or good outcomes in 86.4% of reconstructions, with only 4.2% rated as poor, with rippling and asymmetry being the main limitations identified. Correia-Pinto et al^31^ uniquely combined blinded panel ratings with BREAST-Q, documenting higher cosmesis and lower capsular contracture rates in the PU group compared to ADM.

Overall, the available evidence demonstrates high patient-reported satisfaction and favorable aesthetic outcomes following pre-pectoral reconstruction with PU-coated implants. The main aesthetic drawback was rippling and contour deformity, reported in 12%-19% of cases across several series,^19,26,30^ underscoring the importance of flap thickness and patient selection in optimizing cosmetic results.

## DISCUSSION

Dow Corning Corporation manufactured the first silicone gel breast implant in 1962.^34^ In the 1970s, a second-generation silicone gel implant was introduced, and these new implants were made of silicone, silicone gel, and urethane and were designed to achieve a natural, safe, and pleasing result that the previous prostheses had failed to achieve.^34,35^ Historically, the first attempts at pre-pectoral implant placement in the 1970s were soon abandoned because of unacceptably high complication rates, with reports of implant loss reaching 28%, flap necrosis 13.5%, and capsular contracture as high as 56%. These discouraging outcomes prompted surgeons to seek alternative strategies that could provide more reliable soft-tissue coverage.^36,37,38^ In 1982, Radovan^39^ introduced the 2-stage subpectoral expander–implant technique, in which a tissue expander was placed beneath the pectoralis major muscle and serratus fascia at the time of mastectomy, later exchanged for a permanent implant after expansion. This approach dramatically reduced implant loss, flap necrosis, and contracture rates and became the gold standard in implant-based breast reconstruction for decades.^36^ However, subpectoral reconstruction was not without drawbacks: patients frequently experienced chronic pain, functional impairment due to disruption of the pectoralis major, and breast animation deformity.^36^ The need for 2 surgical stages, with associated hospitalizations and costs, also limited its appeal. An alternative for breast reconstruction was represented by PU-coated breast implants. Early clinical experience with these implants revealed dramatically lower rates of capsular contracture compared to traditional smooth implants.^40,41^ This advantage led to their widespread use through the 1980s.^42^ However, by 1991, safety concerns emerged: it was discovered that the PU foam could degrade into 2,4-toluenediamine (TDA), a compound found to be carcinogenic in rats, prompting a voluntary withdrawal of PU-coated implants from the US market.^42-44^ The search for less invasive alternatives led to the development of 1-stage DTI reconstruction in the 2000s, supported by the introduction of ADM and synthetic meshes.^36^ These devices allowed expansion of the subpectoral pocket to accommodate a definitive implant immediately, without a tissue expander, by reinforcing the inferolateral portion of the muscle.^45^ Acellular dermal matrix use, first reported by Breuing^46^ in 2005 and Salzberg^47^ in 2006, quickly gained popularity, increasing immediate breast reconstruction rates and providing greater aesthetic control. With these innovations and the advent of improved flap assessment techniques, the pre-pectoral approach was reintroduced.^36^ In this modern iteration, the pectoralis major is preserved, and the implant is placed directly beneath the mastectomy flap, often supported with ADMs or PU foam alone. This technique minimizes functional morbidity, eliminates animation deformity, and allows more natural breast shaping.^48^ Meanwhile, subsequent toxicological analyses of PU degradation products demonstrated that the lifetime carcinogenic risk was negligible, and the US FDA concluded that PU-coated implants did not pose a significant cancer risk, recommending no prophylactic removal in asymptomatic patients.^18,34,42^ These findings, combined with the distinct biological and mechanical advantages of PU, facilitated their reintroduction into clinical practice and contributed to the current resurgence of pre-pectoral breast reconstruction.

**Figure 2.**
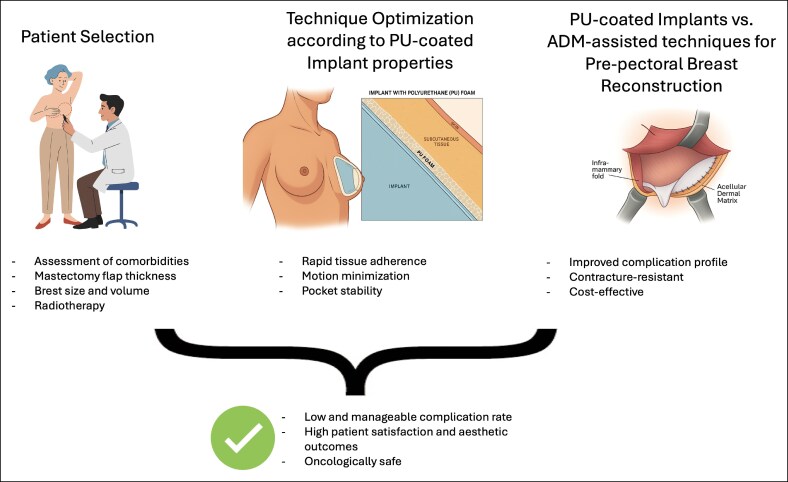
Conceptual framework for the use of polyurethane (PU)-coated implants in direct-to-implant pre-pectoral breast reconstruction. Successful outcomes depend on 3 main factors: (1) careful patient selection; (2) technique optimization tailored to the properties of PU-coated implants; and (3) positioning of PU-coated implants relative to ADM-assisted pre-pectoral reconstruction, highlighting their favorable complication profile, resistance to capsular contracture, and cost-effectiveness. Together, these elements underscore the potential of PU-coated implants as a reliable and efficient option for implant-based breast reconstruction.

With the incremental usage of PU-coated breast implants, a systematic review was necessary to evaluate their use, efficacy, and safety in post-mastectomy DTI pre-pectoral breast reconstruction. This systematic review indicates that this technique is a safe and effective alternative to subpectoral or mesh-assisted techniques. Across the included studies, total complications were infrequent (*n* = 391; 15.63%) and implant loss rates were low (*n* = 24; 0.96%). Minor complication rates were higher but mostly related to manageable cosmetic issues; rippling of the implant edges was the most common minor postoperative complication (*n* = 183; 7.31%), ranging from 0%^23,25,32^ to 21.4%^22^ of cases. Importantly, capsular contracture, a principal concern in implant-based reconstruction, was notably rare (*n* = 26; 1.04%) in the early postoperative period with PU-coated devices.^15,49^ Several studies reported 0 instances of severe (Baker grade III-IV) contracture within the 12-24 months following surgery. The low overall capsular contracture rate observed likely reflects the predominance of short-term follow-up among included studies. Stratification by follow-up duration confirmed that contracture incidence increases over time, consistent with the higher rates reported in long-term cohorts. This finding emphasizes the importance of extended follow-up in future studies to fully assess the durability of PU-coated implants. Patient-reported outcomes were generally favorable as well: postoperative satisfaction scores were high, reflecting the acceptable aesthetic and functional results achieved without muscle disruption.^25,26,28,30-33^ Taken together, these findings support the clinical viability of pre-pectoral PU implant reconstruction, demonstrating low complication rates and good breast form in appropriately selected patients. Bridging this systematic review to present-day practice, the clinical implications that emerge from contemporary evidence are 3-fold: patient selection, technique optimization specific to PU-coated implants biological properties, and where PU-coated implants fit relative to ADM-assisted pre-pectoral reconstruction (Figure 2).

Firstly, careful patient selection emerged as a key factor underpinning the success of this technique. The review and available data emphasize that adequate mastectomy flap thickness and perfusion are essential prerequisites for DTI pre-pectoral placement.^19,26,28,33^ Most authors employed a minimum thickness criterion evaluated through clinical examination or ICG angiography to ensure sufficient soft-tissue coverage over the PU-coated implant. Thinner mastectomy flaps carry a higher risk of ischemia, necrosis, and implant exposure, which can lead to early failure.^50,51^ In patients with borderline flap thickness, strategies such as delayed reconstruction, 2-stage expander use, or reinforcement with autologous tissue may be considered to mitigate risk.^52,53^ The vascularity of the skin envelope is equally crucial: techniques like intraoperative IGC angiography have been utilized to confirm perfusion, especially in NSMs, before committing to an immediate implant in the pre-pectoral plane.^54^

Another consideration is breast morphology. Historically, patients with large or ptotic breasts were often excluded from DTI approaches due to concerns about redundant skin and compromised aesthetics or healing. These cases required a staged reconstruction or a SRM to manage the excess skin. However, emerging evidence suggests that pre-pectoral PU implant reconstruction can be extended to carefully selected patients with moderate macromastia or ptosis.^33^ In one recent study by Salgarello et al^33^ focusing on large, ptotic breasts (mastectomy specimen 400-800 g), immediate reconstruction with pre-pectoral PU implants proved feasible without formal breast-lifting incisions. By tailoring the mastectomy pattern (using a nipple-sparing lateral or radial approach) and assessing flap viability intraoperatively, the patients achieved acceptable outcomes while avoiding additional skin reduction. Overall, complication rates were low (*n* = 12; 13.48%), and no implant losses occurred, demonstrating that even challenging anatomy can be managed with this technique when careful protocols are followed. It should be noted, however, that nearly half of those patients required secondary contour corrections (such as lipofilling) to address issues like rippling or minor deformities. Furthermore, as considered by Lisa et al,^19^ pre-pectoral reconstruction in ptotic breasts not only proved feasible and safe but also appeared to reduce the need for contralateral symmetrization compared with submuscular reconstruction, owing to the possibility of recreating the patient's preexisting ptosis with PU-coated implants.

The impact of radiotherapy must also be carefully considered when selecting candidates for pre-pectoral reconstruction with PU-coated implants. Several included studies^22,23^ excluded previously irradiated patients, reflecting the well-established association between radiotherapy, impaired flap vascularity, and higher rates of wound complications. Other studies such as those by Nebril et al^29^ and Lisa et al^19^ did include patients undergoing post-mastectomy radiotherapy and reported acceptable short-term complication rates, although with a higher incidence of capsular contracture and aesthetic changes during follow-up. Taken together, these findings suggest that while PU-coated implants may mitigate some of the contracture risk compared with other surfaces, radiation therapy, both neoadjuvant and adjuvant, remains a major determinant of long-term outcomes.^55^

Secondly, technical and biological advantages of PU-coated implants must be highlighted. Polyurethane-coated implants provide both mechanical and biological benefits that contribute to their success in the pre-pectoral setting. The PU foam surface promotes rapid adherence to surrounding tissue, which minimizes micromotion and reduces potential dead space.^34^ This property has been associated with lower rates of seroma formation and implant malposition in several studies such as those proposed by de Vita et al,^23^ Salgarello et al,^22^ and Scardina et al^25^ which all reported the absence of postoperative seromas or implant malposition. The same “velcro effect” provides greater pocket stability, which is particularly advantageous in larger or ptotic breasts, where implant displacement is a common concern.^19,33^ On a biological level, PU modifies capsule formation by inducing a fragmented, vascularized capsule with randomly oriented collagen fibers, in contrast to the dense fibrotic capsules seen with smooth or textured implants.^18^ This has translated into consistently lower rates of capsular contracture in both reconstructive and aesthetic settings.^28,29^

Lastly, direct comparisons between PU-coated implants and ADM-assisted reconstructions suggest important differences in both complication profiles and aesthetic outcomes. The studies by Correia-Pinto et al^31^ and Salgarello et al^22^ compare PU-coated implants to the addition of ADMs to assist breast reconstruction with microtextured silicone anatomical implants positioned in pre-pectoral breast reconstruction. Correia-Pinto et al^31^ found that ADM reconstructions were associated with a higher incidence of severe capsular contracture (14.3% vs 0% in PU, *P* = .014), while blinded panel assessments favored ADM in terms of implant visibility and rippling. However, overall cosmesis and BREAST-Q satisfaction were not significantly different after adjustment. By contrast, Salgarello et al^22^ reported a safer complication profile with PU implants, including markedly lower rates of seroma, infection, and severe contracture, along with superior long-term aesthetic ratings on blinded evaluation. The documented lower rates of seroma and infection with PU devices compared to ADM might be likely due to the absence of an interposed biologic scaffold that can harbor fluid or bacteria.^22,27^ Although ADM provides an aesthetic advantage by improving soft-tissue coverage and reducing implant visibility and rippling, especially in thinner patients, these issues in pre-pectoral PU-coated implant reconstructions can be effectively addressed with adjunctive fat grafting.^19,22^ In addition to comparable aesthetic and satisfaction outcomes, PU-coated implants appear to offer procedural and economic advantages over ADM-assisted reconstructions. Although specific costs vary by institution, surgeon, and healthcare setting, PU-based reconstructions are generally more cost-effective due to their single-stage nature, shorter operative time, reduced hospital stay, and absence of adjunctive synthetic or biological materials. Furthermore, the lower incidence of long-term complications may decrease the need for additional imaging, consultations, or reoperations, further contributing to their overall cost-effectiveness.

Based on the available evidence, a set of practical indications for pre-pectoral DTI reconstruction with PU-coated implants can now be delineated. The optimal candidates are adults undergoing immediate NSM or SSM with well-vascularized mastectomy flaps, confirmed both clinically and, when available, by ICG angiography demonstrating complete enhancement within 120 seconds.^26,28,33^ Adequate flap thickness remains fundamental, with most series identifying thresholds of ≥0.8-1.0 cm or an intraoperative mastectomy flap thickness to preoperative breast thickness at the chest wall (I-MFT/*P*-BTC) ratio ≥0.5 to ensure sufficient coverage.^19,26,28,33^ Polyurethane-coated implants are particularly advantageous when eliminating animation deformity and preserving pectoralis function are priorities, and they are especially well-suited for large or ptotic breasts, where their surface adherence facilitates lower-pole shaping and physiologic ptosis, often reducing the need for contralateral symmetrization.^19,22,33^ These devices are most reliably indicated in non-irradiated patients and in situations where avoiding ADM is desirable for cost containment or reduced early seroma risk, while still ensuring low contracture and high satisfaction rates.^23,25,29,31^ Caution is warranted in patients with comorbidities such as diabetes, which has been linked to lower BREAST-Q scores,^32^ and in those receiving post-mastectomy radiation therapy (PMRT), where higher late contracture rates remain a concern.^19,22,31^ In borderline cases, targeted reinforcement or adjunctive fat grafting may broaden indications and mitigate rippling or edge visibility while maintaining the advantages of the pre-pectoral PU approach.^26,28,30^

Clinically, these findings position PU-coated implants as a valuable alternative to ADM-based pre-pectoral reconstruction. While current evidence is encouraging, it is derived largely from retrospective, short-term studies; prospective multicenter trials with extended follow-up are needed to define the durability of these outcomes, the role of PU implants in irradiated or high-risk populations, and their comparative cost-effectiveness. Until then, PU-coated implants should be considered an evidence-supported option in immediate pre-pectoral reconstruction, offering the potential to enhance safety, reduce contracture, and deliver stable, aesthetically pleasing results for appropriately selected patients.

### Limitations

Our study has different limitations. Most included studies were retrospective, single-center cohorts with inherent risks of selection bias, as patients were often carefully chosen for favorable anatomy and healthy mastectomy flaps. Reporting was heterogeneous: some cohorts excluded smokers, patients who performed radiation therapy, or women with voluminous breasts, while others included them under stricter protocols, limiting comparability. Furthermore, several studies did not report on primary patient data such as follow-up period, patient comorbidities, tumor histology and stage, and neo-adjuvant or adjuvant treatments. Follow-up was generally short to mid-term (often 12-36 months), which constrains the evaluation of late and long-term complications such as capsular contracture, implant rupture, or long-term aesthetic changes. Outcome reporting was also inconsistent, while some studies used validated PROMs such as the BREAST-Q or blinded panel evaluations, others relied on non-validated surveys or surgeon impressions, complicating pooled interpretation. Finally, only 2 comparative studies directly evaluated PU-coated implants against ADM-assisted reconstruction, underscoring the need for more robust head-to-head evidence.

### Future Studies

Few studies directly compared PU-coated implants with ADM- or mesh-assisted pre-pectoral reconstructions. The limited availability of comparative data precluded a head-to-head analysis in this review. Future research should prioritize well-designed, prospective, and ideally multicenter studies comparing PU-coated implants to ADM and other reconstruction strategies, with standardized inclusion criteria and outcome reporting. Particular attention should be paid to high-risk populations, such as irradiated patients, smokers, and women with macromastia or thin mastectomy flaps, where data remain limited but clinically relevant. Longer-term follow-up is essential to determine whether the early protection against capsular contracture observed with PU persists beyond 5-10 years. Moreover, adjunctive strategies such as routine or prophylactic fat grafting should be formally studied to clarify their role in minimizing rippling and optimizing cosmesis. Finally, cost-effectiveness analyses and patient-centered outcome measures will help define the broader clinical value of PU implants compared with ADM, especially in healthcare systems where resource allocation is a major concern.

## CONCLUSIONS

This is the first systematic review that demonstrates that DTI pre-pectoral breast reconstruction with PU-coated implants is a safe and effective technique, associated with low complication rates and high patient satisfaction. The unique surface properties of PU confer biological and mechanical advantages, allowing stable pocket control without the need for ADM coverage. Importantly, successful results depend on rigorous patient selection, being meticulous on adequate flap thickness and perfusion, risk factor and comorbidity assessment, and optimal surgical planning.

## Supplemental Material

This article contains supplemental material located online at https://doi.org/10.1093/asjof/ojaf162.

## Supplementary Material

ojaf162_Supplementary_Data
